# Towards a new paleotemperature proxy from reef coral occurrences

**DOI:** 10.1038/s41598-017-10961-3

**Published:** 2017-09-05

**Authors:** Andreas Lauchstedt, John M. Pandolfi, Wolfgang Kiessling

**Affiliations:** 1 GeoZentrum Nordbayern, Paleobiology, Erlangen, 91054 Germany; 2 The University of Queensland, ARC Centre of Excellence for Coral Reef Studies and School of Biological Sciences, Brisbane, QLD 4072 Australia

## Abstract

Global mean temperature is thought to have exceeded that of today during the last interglacial episode (LIG, ~ 125,000 yrs b.p.) but robust paleoclimate data are still rare in low latitudes. Occurrence data of tropical reef corals may provide new proxies of low latitude sea-surface temperatures. Using modern reef coral distributions we developed a geographically explicit model of sea surface temperatures. Applying this model to coral occurrence data of the LIG provides a latitudinal U-shaped pattern of temperature anomalies with cooler than modern temperatures around the equator and warmer subtropical climes. Our results agree with previously published estimates of LIG temperatures and suggest a poleward broadening of the habitable zone for reef corals during the LIG.

## Introduction

More than other marine organisms, reef corals are considered to be sensitive to current global warming and associated stressors^1–5^. Temperature is probably a dominant factor controlling the latitudinal distribution of reef corals although a suite of additional factors may be involved^6–8^.

Although the latitudinal extent of tropical reefs shows no correspondence with global temperature changes on geological time scales^9^, there are several examples of poleward reef coral range-shifts with Pleistocene-Holocene warming episodes^10–13^. Just as marine organisms in general^14, 15^, coral distributions thus seem to track temperature changes. The response of reef corals to climate change is variable among taxa and traits^7, 16–20^ but multivariate approaches may permit linking changes of coral distributions with temperature changes. Reconstructing past temperatures from fossil assemblage composition is common practice for Pleistocene microfossil assemblages^21–23^. The reconstructions rest on the basic assumption that the composition of faunal assemblages is driven by the physical nature of the surrounding water.

Here we apply the micropaleontological toolkit for paleoclimate reconstructions to reef corals in order to constrain tropical to subtropical temperatures of the Last Interglacial (LIG) episode (ca. 125 kyr), for which micropaleontological and geochemical proxy data are still limited^24, 25^. We use the geographic distribution of reef coral assemblages in 1° geographic grid cells to derive models of modern mean sea-surface temperature (SST) and seasonal temperature variability (STV). The best-fit model is applied to LIG coral occurrences to reconstruct SST and STV anomalies along latitudinal gradients. Lastly, we compare the modeled latitudinal temperature gradients of the LIG with published proxy data and independently developed climate models.

## Results

There are several significant correlations between modern reef-coral occupancy and mean annual SST (see the Supplementary Information, Table S1). Negative correlations prevail suggesting that the occupancy of most genera tends to decrease with increasing SST. Only members of the *Acroporidae*, *Agariciidae*, *Fungiidae, Mussidae* and *Merulinidae* show positive correlations.

SSTs derived from transfer functions correspond well with measured temperature data. Using an Artificial Neural Network (ANN) approach, the median temperature deviation between observed and modeled SST is −0.297 °C and lower near the equator than in the subtropics (Fig. S1). Results are virtually independent of taxonomic level (genus or species) and the method to derive transfer functions (ANN or factor analysis) (Fig. S2).

**Figure 1:**
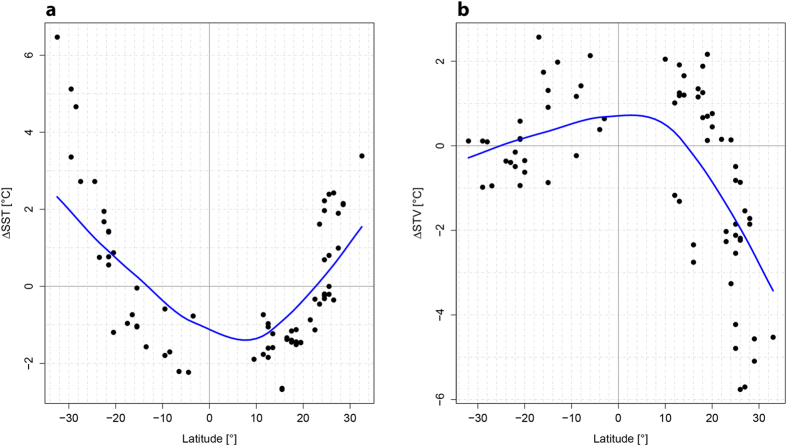
Modeled mean annual sea-surface temperatures, SST (**a**) and seasonal temperature variability, STV (**b**) anomalies between the LIG and the Recent plotted along latitudes. Modeled values are derived from an artificial neural network (ANN) approach of proportional reef coral species occurrence data in 1° grid cells against mean annual SSTs. Blue line is LOESS regression line (degree of smoothing = 0.8).

Results at both the species and genus level suggest 1 °C cooler inner tropical (0–15° N/S) and 2 °C warmer subtropical (>25° N/S) mean SSTs in both hemispheres during the LIG than today (Fig. 1a, Fig. S2). LIG temperature seasonality is reconstructed to have been much lower in the subtropical northern hemisphere than today (Fig. 1b), whereas tropical (0–20° N/S) seasonality was slightly elevated in both hemispheres. Southern hemisphere seasonality was virtually unchanged in the subtropics.

Model performance worsens systematically with a decrease of the species pool and the number of assemblages used for model creation (Fig. S3). However, the mean total temperature deviation between the LIG and the modern is modest (0.1–0.5 °C) even with a small fraction of the actually sampled species and grid cells that are used to inform our model.

**Figure 2:**
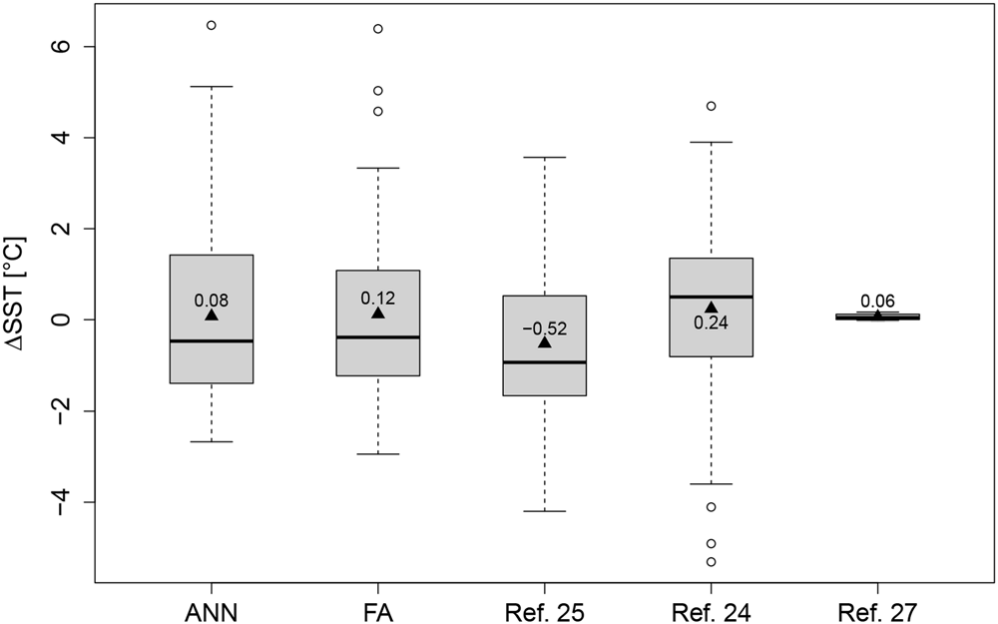
Boxplot of modeled mean annual SST anomalies (ANN and FA) based on corals and published temperature data^24, 25, 27^ between the LIG and the Recent. Straight lines in the boxes show the median temperature from 32°S to 33°N, triangles and numbers indicate the mean.

**Figure 3:**
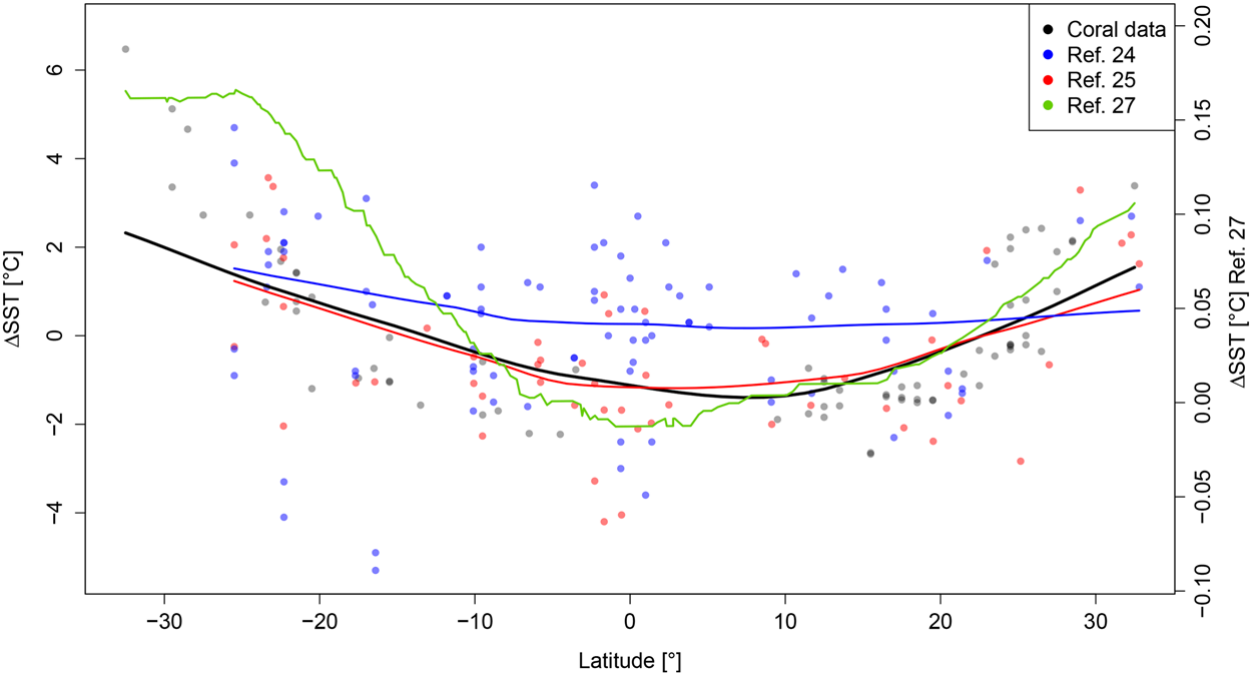
Plot of mean annual SST anomalies between the LIG and the Recent from 32°S to 33°N, independent proxy data^24, 25^ and values from a climate model^27^. Lines represent LOESS regression (span = 0.8). The correlations between values of the ANN anomalies and independent data are all significant if they are binned within 5° latitudinal bands (see Table 1, Fig. S4).

Various geochemical and faunal proxy data have been compiled for SSTs of the LIG^24–26^ and we also consider the LOVECLIM climate model reporting LIG anomalies along latitudinal gradients^27^. Proxy compilations of marine data within the LIG coral reef zone (32°S to 33°N), show small positive as well as negative SST anomalies in the LIG, with global averages of −0.52 °C^25^ and + 0.24 °C^24^. All proxy data and the coral-modeled anomalies have statistically indistinguishable means (ANOVA test: all *p* > 0.1) (Fig. 2). The medians of coral-derived temperature anomalies are within the range of those from other proxy data and are slightly higher than the climate model, but not significantly so in pairwise comparisons (Wilcoxon signed ranks test: *p* > 0.1, throughout, except for the combination ANN on species level with the results of ref.^27^, for which *p* = 0.011). Our results are also spatially correlated with the proxy data in McKay^24^ and the climate model^27^ (Fig. 3). Due to a limited spatial coverage of the proxy data, we tested the correlations in 5° latitudinal bands (Table 1, Fig. S4). The LOVECLIM climate model yields much less variance, suggesting the same temperature as today near the equator and only slightly higher temperatures in the subtropics and high latitudes (Fig. 3). Other model-based temperature reconstructions and model intercomparisons for the LIG exist^25, 28, 29^. Modelled temperatures within a 30° S/N band indicate 2 °C cooler to 2 °C warmer temperature anomalies, depending on the applied model, and season^28^. Lunt, *et al*.^29^ also find slightly cooler equatorial and increased extratropical temperature anomalies, although for many oceanic regions the different climate models applied show less than 70% agreement on the sign of temperature change. All approaches (see also ref. 25) agree in the overall shape of latitudinal temperature anomalies, showing a robust pattern of lower than modern equatorial temperatures and higher than modern subtropical temperatures (Fig. S2).

**Table 1:** Correlation matrix of modeled LIG temperature anomalies with published faunal and geochemical temperature proxy and LOVECLIM climate model data.

	species ANN	genera ANN	species FA	genera FA	Ref. 25 all data	Ref. 25 annual	Ref. 25 faunal	Ref. 25 chemical	Ref. 24 all data	Ref. 24 annual	Ref. 24 faunal	Ref. 24 chemical	Ref. 27
species ANN		0	0	0	0.009**	0.013*	0.009**	0.007**	0.033*	0.006**	0.627	0.054	<0.001***
genera ANN	0.93		0	0	0.02*	0.042*	0.02*	0.03*	0.02*	0.108	0.187	0.008**	0.005**
species FA	0.96	0.95		0	0.036*	0.058	0.036*	0.042*	0.019*	0.029*	0.328	0.013*	0.005**
genera FA	0.97	0.94	0.99		0.028*	0.058	0.028*	0.042*	0.031*	0.022*	0.489	0.043*	0.002**
Ref. 25 all data	0.71	0.66	0.61	0.63		0	0	<0.001***	0.15	0.006**	0.77	0.259	0.005**
Ref. 25 annual	0.78	0.68	0.65	0.65	0.94		0	0	0.244	0.003**	0.61	0.112	0.005**
Ref. 25 faunal	0.71	0.66	0.61	0.63	1	0.94		<0.001***	0.15	0.006**	0.77	0.259	0.005**
Ref. 25 chemical	0.82	0.72	0.68	0.68	0.88	0.96	0.88		0.2	0.013*	0.911	0.03*	0.006**
Ref. 24 all data	0.62	0.66	0.66	0.62	0.42	0.41	0.42	0.44		0.096	0.001***	0.007**	0.344
Ref. 24 annual	0.79	0.54	0.68	0.71	0.76	0.87	0.76	0.78	0.53		0.606	0.089	0.17
Ref. 24 faunal	0.18	0.45	0.35	0.25	0.1	−0.21	0.1	−0.05	0.84	−0.2		0.088	0.979
Ref. 24 chemical	0.62	0.78	0.75	0.65	0.37	0.57	0.37	0.72	0.75	0.54	0.6		0.979
Ref. 27	0.81	0.73	0.73	0.77	0.73	0.81	0.73	0.79	0.29	0.45	0.01	0.01	

Spearman-rank order correlations among proxy data and modeled sea-surface temperature anomalies across 5° latitudinal bands (means). Correlation coefficients are reported in the lower left, p-values in the upper right. Asterisks indicate degree of significance, ***<0.001, **<0.01,*≤0.05. ANN = artificial neural networks; FA = factor analysis.

Few independent proxy data on temperature seasonality exist^24, 30–33^. Patterns derived from Caribbean coral geochemistry suggest higher peak LIG temperature seasonality than in the modern^33^, which is opposite to our inference. Other coral-derived temperatures from the same region indicate a similar than today late LIG temperature seasonality anomaly at 12.5°N^31^ which is very close to our mean calculated anomaly at 12°N (~ −0.1 °C). Also other studies on northern hemisphere temperature seasonality coincide with their results of higher than modern temperature seasonality anomalies in the peak LIG (~126 and 122 ka)^30, 32^ and thus also contrast our findings.

## Discussion

Our occurrence-derived coral temperature trends match both independent proxy data and climate models for the LIG. The approach of reconstructing temperature from reef coral occurrence data thus appears feasible. Reconstructed temperature seasonality anomalies, on the other hand, contradict proxy derived seasonalities as well as changes in insolation seasonality predicted from orbital forcing. It is documented for the northern hemisphere, that seasonality during the LIG underwent a major evolution, from higher anomalies during the peak LIG^33^ to near modern values in the late LIG^31^. Thus, our results on temperature seasonality may be biased by time averaging, because coral occurrence data simply cannot be resolved stratigraphically to a distinct episode within the LIG. Although the temporal resolution of coral assemblage data cannot be as fine as that of microfossil and geochemical proxies^25^, they may serve as good temperature proxies for coastal areas where microfossil proxies are scarce. Microfossil temperature estimates are calibrated from species assemblages at the top of drill cores (comprising the last 100–500 years) and their correspondence with local temperature records at the drill localities. Characteristic assemblages are then traced back through time (in the core), providing indirect temperature estimates of former time periods. In our approach, individual samples used for microfossil temperature estimates had to be replaced with coral assemblages over larger geographic areas (1° grid cells), resulting in more spatially averaged temperature estimates but with an improved spatial coverage. We therefore emphasize the potential for SST anomalies to explain previously observed range shifts in the LIG^11^.

Interglacial and post-glacial poleward coral range shifts are often reported^10–13, 34^ and attributed to global or regional warming. While most previous studies noted range expansions, Kiessling *et al*.^11^ noted similarly strong poleward movements of the trailing (equatorward) range edges as of the leading (poleward) edges. With our new results, trailing edge movements correspond with cooler than modern SST while leading edge movements are associated with intensified warming relative to the modern. Even though equatorial temperatures were potentially cooler than today, last interglacial temperatures resulted from a massive global warming after MIS 6^35^. Thus, peak interglacial SSTs could have exceeded the temperature range to which corals were previously adapted. There is ongoing debate about which factors influence coral reef success in the aftermaths of glacial maxima in times of progressing climate warming.^36, 37^ There is agreement that postglacial temperature rise or climate variability has not affected reef growth^38, 39^. Coral community changes from glacial to interglacial reefs are only beginning to be explored but one study demonstrated significant differences in coral community structure between climate regimes in Huon Gulf, Papua New Guinea^40^. Although this study emphasized regional factors to explain the changes, the results do not contradict climate-induced range shifts. Consequently, we argue that even cooler than modern equatorial temperatures triggered trailing-edge shifts of reef corals in the LIG. This may imply that the migration of corals was less dependent on the absolute temperature level than on the pace of warming^41^.

With respect to temperature seasonality anomalies a pronounced asymmetry between the northern and southern hemisphere, with lowered northern hemisphere temperature seasonality and similar to modern southern hemisphere temperature seasonality, is suggested by the coral model. This finding contradicts other proxy and model based estimates of LIG temperature seasonality, which propose higher temperature seasonality anomalies in the northern hemisphere at the peak LIG and equal to modern temperature seasonality anomalies in the late LIG^30–33^. We attribute this discrepancy to time averaging, inevitably involved in coral community studies. In addition, coral communities are stable in their assemblage composition over longer time intervals^42^. Therefore, thermal signals, modelled by coral occurrence data, are affected by this slow response, as well as by time averaging. Longer-term stability of reef coral assemblages thus acts similar as time averaging because it also hampers finer scale temporal resolution of environmental signals. Consequently, time averaging is not only an impediment in the data but mirrors partly the nature of longer-term reef coral assemblage behavior. Therefore it is difficult to compare our results with data that are temporarily finer resolved.

Accepting the previously published evolution of temperature seasonality, seasonality should have been higher than today in the northern hemisphere for most of the LIG. Seasonality anomalies may have approached 4–5 °C at ~12°N^33^ and 8.4 °C at ~30°N^30^ in the mid LIG. Higher seasonality was probably a result of winter cooling with a small degree of summer warming in the earlier LIG (~127ka)^32^. Absolute minimum temperatures by intensified winter cooling may have been mitigated by overall warmer temperatures. This mitigation could also explain the leading edge movements because thermal habitat area would expand latitudinally.

In conclusion, we propose higher than modern temperatures in mid latitudes accompanied with lower equatorial temperatures during the LIG, which is consistent with proxy and model data. This might account for the observed range shifts in reef corals. As occurrence based temperature seasonality anomaly estimates are contradicting independent proxy data, presumably caused by time averaging, our method may not be suitable for reconstructions of LIG temperature seasonalities. Nevertheless, reef coral occurrence data, although time-averaged, have potential to develop into a new paleoclimate temperature proxy for the Pleistocene.

## Methods

### Data

Occurrence data of zooxanthellate corals were downloaded from two comprehensive databases: OBIS (www.iobis.org) for recent occurrences and the Paleobiology Database (PaleoDB, www.paleobiodb.org) for fossil ones. Azooxanthellate and apoxanthellate corals were excluded. Additionally, a depth filter was applied to only include corals within the uppermost 60 m of the water column. Fossil data were filtered to only include species occurrences from the Last Interglacial (LIG). The final datasets consists of 299,676 recent and 2,573 fossil coral occurrences. Taxonomy was corrected using the World Register for Marine Species (WoRMS; www.marinespecies.org).

Sea-surface temperature (SST) data were gathered from the Hadley Center (www.metoffice.gov.uk) with a 1° grid resolution and mean monthly values for the years 2006–2016 (2012 excluded). For each grid cell, mean SST and seasonal temperature variability (STV) were calculated. STV was defined as the absolute annual temperature range, the temperature variability from the coldest to the warmest month.

Supplementary data from PaleoDB and OBIS are available for download at PANGAEA (https://doi.pangaea.de/10.1594/PANGAEA.879342).

### Analyses and Sensitivity Tests

We calculated the proportions of coral genera and species in 1° grid cells. Cells occupied by just one genus were excluded to avoid bias by taxonomic foci (e.g., intense monitoring of *Acropora* in the Caribbean or extensive sampling of fossil *Porites* for radiometric age dating).

Two multivariate approaches were applied to retrieve a model of SST and STV. Both approaches used the proportional occurrence data of extant coral taxa, which also have a fossil record in the LIG (187 species, 66 genera). In our fist approach, we used transfer functions derived from factor analyses as implemented in the R package “rioja”^43^. Transfer functions were initially developed by Imbrie and Kipp^21^ to predict environmental variables using foraminiferal assemblages. The resulting model was tested against observed values of SST and then applied to Pleistocene coral occurrence data within the same 1° grids to model temperature and temperature seasonality. As the resulting model equation ideally requires all genera to have greater than zero occupancy and the Pleistocene occurrence data is sparse in terms of total amount as well as spatial coverage compared to the recent, we omitted grids with less than two sampled genera.

In our second approach we used Artificial Neural Networks (ANN) implemented in the R package “nnet”^44^. We used 5 hidden layers, a possible maximum of 100000 iterations and 1000 weights for species and 5 hidden layers with 100000 iterations and 400 weights for genera. Weights represent the strength of the connection between two nodes in an ANN. Hidden layers are the number of additional node instances between the input (coral occurrence data) and output layer (SST). First we tested the method internally to predict recent SST with recent coral data. We then used recent data as a training dataset and Pleistocene occurrence data for predicting former SST. All calculations were then iterated 1000 times and we took the calculated median values as the final result. All calculations and analyzes were performed within the statistical environment of R^45, 46, 47^.

Further information and full methodical description can be found in the Supporting Information (SI).

## Electronic supplementary material

Supplementary Information
