# Supplementary material for: Towards a new paleotemperature proxy from reef coral occurrences
Source: Sci Rep. 2017 Sep 5;7:10461. doi: 10.1038/s41598-017-10961-3 (PMC5585234; doi:10.1038/s41598-017-10961-3)
Supplement: Supplementary file 1 — Supplementary Information [file 41598_2017_10961_MOESM1_ESM.pdf]

Supporting Information

Towards a new paleotemperature proxy from reef coral occurrences

Andreas Lauchstedt, John. M. Pandolfi and Wolfgang Kiessling

SI Data and Methods

OBIS data were downloaded on February 11, 2016 and comprised 310,127 occurrences of 781 species in 168 genera, PaleoDB data were downloaded on February 23, 2016 and covered late/upper Pleistocene 2666 occurrences of scleractinian corals. Occurrence data from the Paleobiology Database (PBDB) (https://paleobiodb.org) were downloaded with the criteria “Scleractinia” and “Pleistocene”. These data were filtered to keep only last interglacial occurrences. Data labelled “last” or “latest Pleistocene”, “MIS5e” or directly dated occurrences within the time interval 120-130ka were included in the analyses and were further called LIG occurrences. Coral and sea surface temperature (SST) data were gridded using the R package “raster”[1]. All further analyses were performed in the R programming environment applying the functions and packages detailed below.

(i) Treatment of data

Coral occurrences were aggregated into 1°x1° grid cells. For each grid cell we calculated the proportional occurrences of all extant and fossil species and genera. These data fed the factor and artificial neural network analyses. The proportions of coral species and genera in each grid cell add up to 1. Temperature data were linked to each occupied grid cell of the recent data.

(ii) Factor analysis, artificial neural networks and sensitivity tests.

Factor analysis and artificial neural networks were performed using the R package “rioja”[2] and the package “nnet”[3] with the functions “IKFA” and “nnet”. Before performing the factor analysis the appropriate number of factors was determined with the “fa.parallel” function in the “psyche” package[4]. In total, we used 22 factors. We

performed this analysis on genus and species level using all Pleistocene still extant species (187) and genera (66). No taxonomic or latitudinal constraints were set but we used a minimum of at least 2 species/genera present in a grid as a prerequisite for the Recent as well as the Pleistocene data. In order to test the sensitivity of the modeled recent temperature within 1° grids we calculated the error in °C our model produced with the recent data (Fig. S1; the map was created with the World Borders Dataset: www.thematicmapping.org). As a second test we created multiple latitudinal and taxonomic subsets of recent data. We modeled Pleistocene - Recent temperature difference and % deviation by using random subsets of 40, 60, 80, 100, 130 ,160 and 180 species and 35, 40, 45, 50, 55 and 60 genera as well as 40, 60, 80, 100, 130, 160, 180 and 220 grid cells out of 287 in total, respectively. For each subset we ran 1000 iterations and calculated the mean temperature and temperature seasonality for each grid cell. We determined the total and percentage deviation of these results from the analysis comprising all species/genera (example on species level with spatial and taxonomic constraints: Fig. S3).

(iii) Correlation tests with climate models and proxy data

We used Spearman`s rank correlation to test for similarities of the modeled coral data and independent reports of temperatures during the LIG (Table 1). McKay, et al. [5] provide their data as pdf files in the supporting information. McKay, et al. [5] provide information on the kind of proxy (faunal and geochemical). The data of Yin and Berger [6] is not accessible online. We extracted the Δ SST values for the LIG by using the Web Plot Digitizer on their figure 7b. The data from Hoffman, et al. [7] is accessible online, and we extracted the mean temperature information for the time period from 120-129 kyr. For correlation tests, all proxy data that was derived from factor analysis, mostly of microfossils, was labelled faunal, all geochemical proxies chemical. Correlation analyses were performed using a 5° latitudinal gridding and taking the mean.

References

1 Hijmans, R. J. *et al.* Package 'raster'. *R package* (2015).

2 Juggins, S. rioja: Analysis of Quaternary science data. *R package version 0.5–6* (2009).

3 Ripley, B. & Venables, W. nnet: Feed-forward neural networks and multinomial log-linear models. *R package version* **7** (2011).

4 Revelle, W. & Revelle, M. W. Package 'psych'. (2016).

5 McKay, N. P., Overpeck, J. T. & Otto-Bliesner, B. L. The role of ocean thermal expansion in Last Interglacial sea level rise. *Geophys. Res. Lett.* **38** (2011).

6 Yin, Q. Z. & Berger, A. Individual contribution of insolation and CO2 to the interglacial climates of the past 800,000 years. *Climate Dynamics* **38**, 709-724, doi: 10.1007/s00382-011-1013-5 (2012).

7 Hoffman, J. S., Clark, P. U., Parnell, A. C. & He, F. Regional and global sea-surface temperatures during the last interglaciation. *Science* **355**, 276-279, doi:10.1126/science.aai8464 (2017).

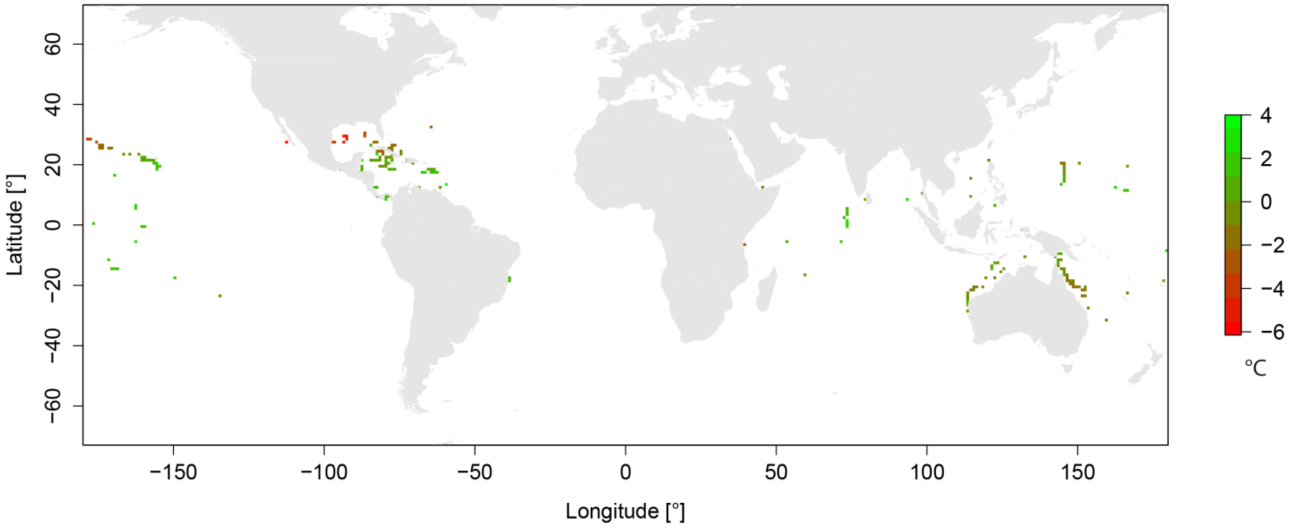

Figure S1: Temperature deviation created by modelling recent temperature data within 1° grid cells and subtracting real temperature values. Legend shows deviation in °C. The shape file for the world borders was downloaded at: http://thematicmapping.org/downloads/world_borders.php.

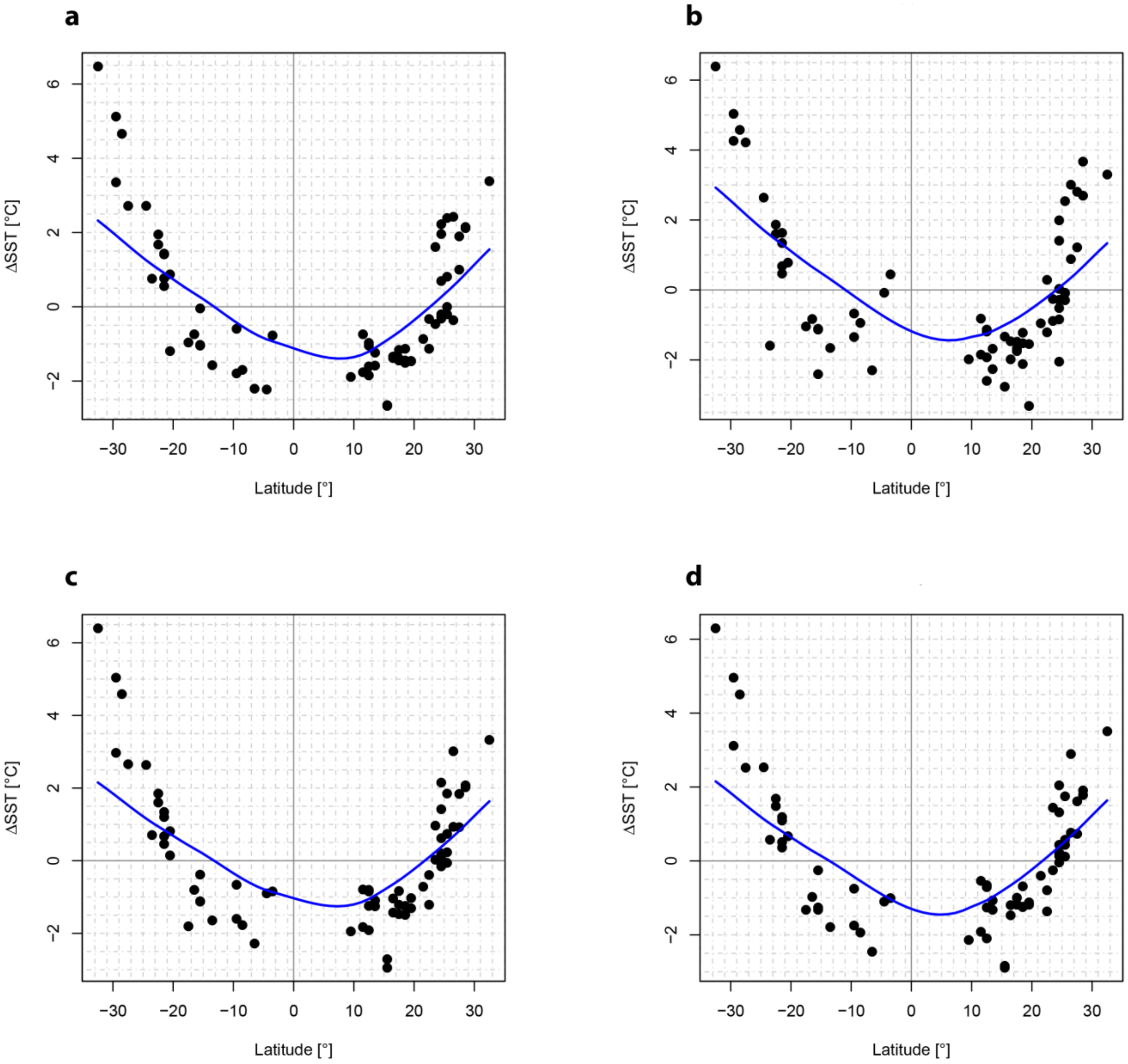

Figure S2: Modeled mean annual temperature anomaly between the LIG and the Recent for different latitudes. Modeled values are derived from proportional coral occurrence data in 1° grid cells against mean annual sea-surface temperatures on species (a, c) and genus level (b, d) applying artificial neural networks (a, b) and factor analysis (c, d). LOESS regression lines (span= 0.8) in blue.

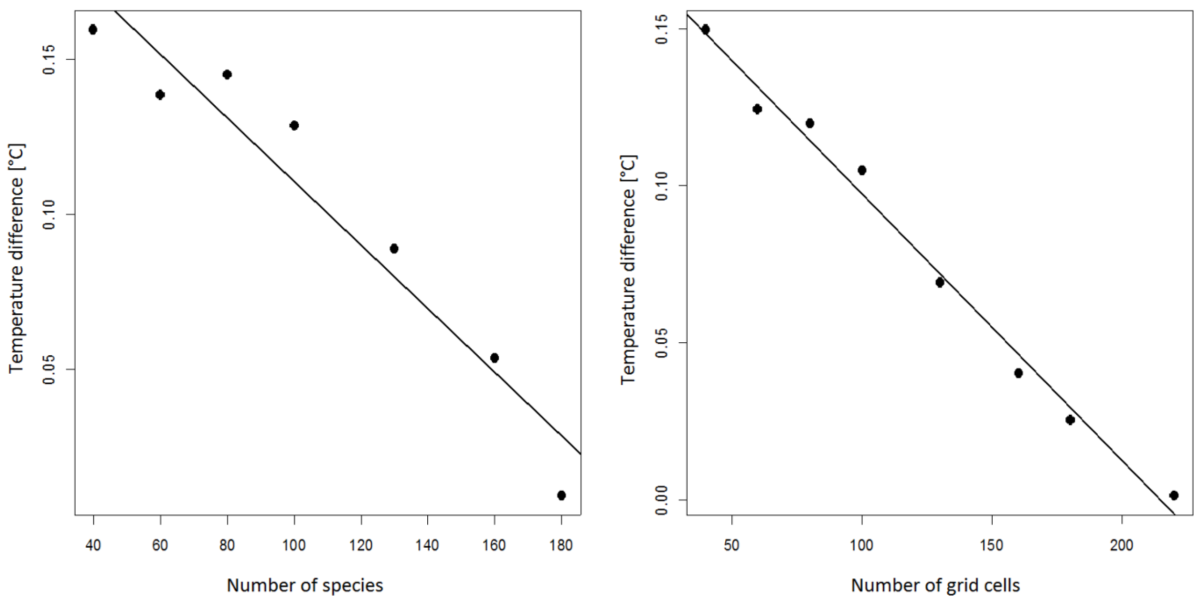

Figure S3: Difference of transfer function modeled temperatures between the whole dataset and subsets comprising different numbers of species (left) and grid cells (right) included in the calculations.

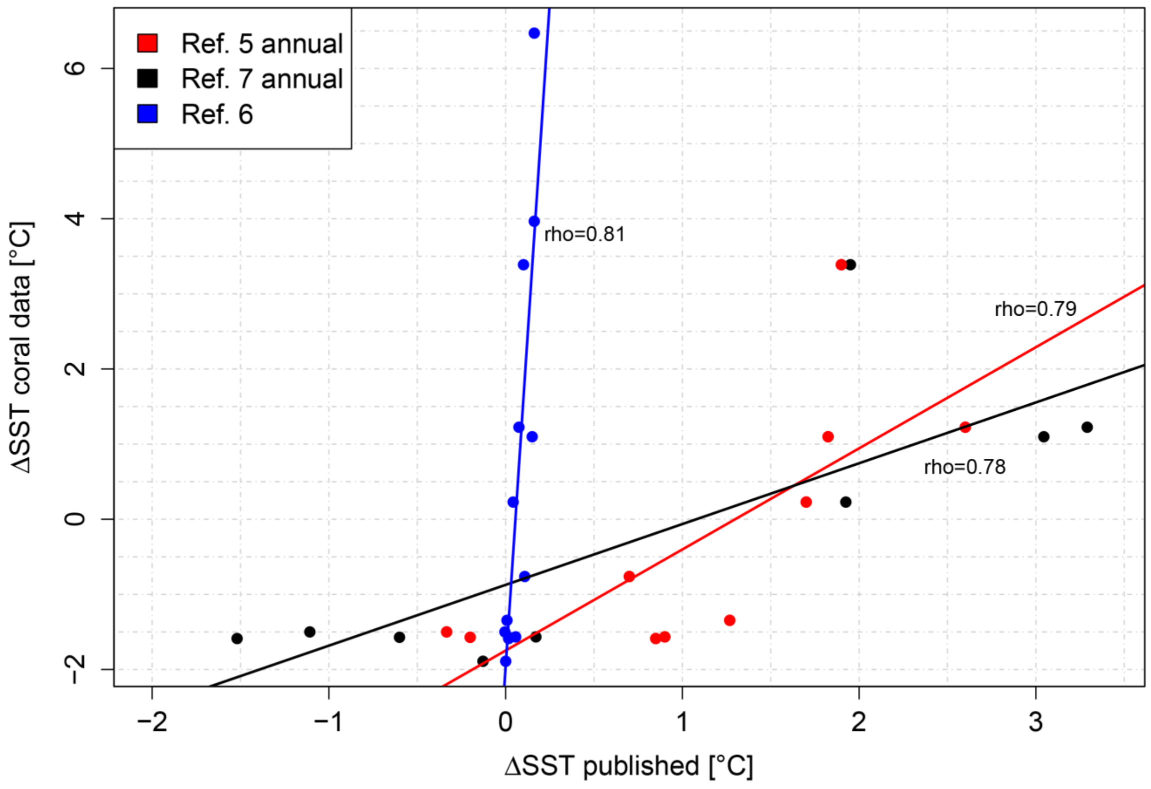

Figure S4: Scatterplot of mean annual SST anomalies between the LIG and the Recent from 32°S to 33°N against independent proxy data[5,7] and values from a climate model [6]. Data were aggregated to 5° latitudinal bands. The correlations between the ANN anomalies and independent data are all significant: Annual means in the McKay et al. data[5] = ref. 24 in the main text: p = 0.006; annual means in the Hoffman et al. data[7] = ref. 25 in the main text: p = 0.013; Yin and Berger model results[6] = ref. 27 in the main text : p < 0.001. Straight lines are linear regression lines. Correlation coefficients are shown in Table 1.

Correlations between recent coral genera and families with mean annual sea surface temperature (SST) and seasonal temperature variability (STV).

Family	Genus	Correlation with SST	Significance	Correlation with STV	Significance
Acroporidae	*Acropora*	-0.22	*	0.26	**
Acroporidae	*Alveopora*	-0.44	*		
Acroporidae	*Astreopora*	0.34	*	-0.35	*
Acgariciidae		0.23	**		
Acgariciidae	*Leptoseris*	0.29	*		
Acgariciidae	*Pavona*	0.33	**	-0.24	*
Astrocoeniidae		-0.38	***	0.42	***
Astrocoeniidae	*Stephanocoenia*	-0.51	***	0.52	***
Fungiidae		0.31	**		
Fungiidae	*Pleuractis*			0.64	*
Fungiidae	*Cycloseris*	0.34	*		
Fungiidae	*Herpolitha*	-0.43	*		
Lobophyllidae		-0.56	***	0.54	***
Lobophyllidae	*Acanthastrea*	-0.45	**		
Lobophyllidae	*Echinophyllia*	-0.33	*		
Lobophyllidae	*Lobophyllia*	-0.56	***	0.55	***
Merulinidae		0.18	*		
Merulinidae	*Paragoniastrea*	-0.8	***		
Merulinidae	*Dipsastraea*			0.4	**
Merulinidae	*Coelastrea*	-0.7	***		
Merulinidae	*Echinopora*			0.36	*
Merulinidae	*Favites*	-0.29	*	0.32	*
Merulinidae	*Platygyra*			0.43	**
Merulinidae	*Merulina*	-0.44	*	0.54	**
Merulinidae	*Pectinia*	0.43	*		
Mussidae		-0.3	***	0.29	**
Mussidae	*Favia*	0.29	*		
Mussidae	*Montastraea*	-0.25	*		
Mussidae	*Isophyllia*	-0.6	***	0.61	***
Mussidae	*Manicina*	-0.75	***	0.67	***
Mussidae	*Mussa*	-0.75	***	0.78	***
Mussidae	*Scolymia*	-0.64	***	0.6	***
Poritidae	*Goniopora*			0.34	*
Meandrinidae		-0.51	***	0.39	***
Meandrinidae	*Dichocoenia*	-0.62	***	0.52	***
Meandrinidae	*Eusmilia*	-0.34	*		
Meandrinidae	*Meandrina*	-0.49	***	0.3	*
Siderastreidae	*Pseudosiderastrea*	-0.83	*		

Table S1: Spearman rank correlations of 1° gridded coral occurrence data on family and genus level derived from OBIS with mean annual SST and mean annual STV in 2006-2016. Only taxa with at least one significant correlation are shown. Asterisks indicate p-values: * <0.05, **≤0.01, *≤0.001.**
